# Axis-dependent relationships and agreement between whole-body and limb-specific center of pressure measurements in dogs

**DOI:** 10.3389/fvets.2026.1958312

**Published:** 2026-09-03

**Authors:** Christiane Lutonsky, Nadja Affenzeller, Julia Wegscheider, Masoud Aghapour, Christian Peham, Barbara Bockstahler

**Affiliations:** 1 Section of Physical Therapy and Rehabilitation, Clinical Unit of Small Animal Surgery, Clinical Centre for Small Animal Health and Research, Clinical Department for Companion Animals and Horses, University of Veterinary Medicine, Vienna, Austria; 2 Behavioural Medicine, Clinical Unit of Small Animal Internal Medicine, Clinical Centre for Small Animal Health and Research, Clinical Department for Companion Animals and Horses, University of Veterinary Medicine, Vienna, Austria; 3 Movement Science Group, Clinical Centre for Equine Health and Research, Clinical Department for Small Animals and Horses, University of Veterinary Medicine, Vienna, Austria

**Keywords:** canine posturography, center of pressure, measurement agreement, postural sway, base of support

## Abstract

Static posturography is widely used to assess postural stability in dogs. Considerable methodological variation exists regarding whether center of pressure (COP) parameters are derived from whole-body, forelimb-, or hindlimb-based measurements and whether values are expressed as absolute or base of support (BOS)-normalised parameters. The aims were to examine the associations between whole-body, forelimb-, and hindlimb-based COP measurements in standing dogs and to evaluate the effect of BOS normalisation on agreement between these measurement approaches. Static posturography was performed in clinically healthy adult dogs using a pressure measurement plate. Craniocaudal displacement, mediolateral displacement, support surface, statokinesiogram length, and average speed were analysed using whole-body, forelimb-, and hindlimb-based COP calculations and BOS-normalised parameters. Associations between measurement approaches were assessed using Spearman’s rank correlation coefficients, while agreement was evaluated using Bland–Altman analyses. Positive correlations were observed between measurement approaches, although their strength and significance varied among COP parameters. Mediolateral displacement demonstrated stronger associations between measurement approaches than craniocaudal displacement, while statokinesiogram length, average speed, and support surface showed moderate to strong correlations across most comparisons. However, Bland–Altman analyses revealed systematic differences and, for several parameters, magnitude-dependent patterns, indicating limited agreement despite significant correlations. BOS normalisation altered the magnitude of several COP parameters but did not consistently improve agreement or eliminate systematic differences between measurement approaches. These findings demonstrate that whole-body, forelimb-, and hindlimb-based COP measurements quantify related but not interchangeable aspects of postural stability. Consequently, measurement approach should be considered when interpreting stabilographic outcomes and comparing results across studies.

## 1 Introduction

Static posturographic measurements are widely applied in canine research to quantify postural stability (1–17). These measurements are based on the analysis of the center of pressure of the body (COP), defined as the point location of the resultant ground reaction force vector acting between the limbs and the supporting surface (1, 2). The movement of COP reflects the continuous neuromuscular adjustments required to maintain an upright stance, and analysis of COP trajectories therefore provides insight into postural sway and the underlying control strategies of the neuromuscular system (3).

Veterinary researchers use these measurements to assess the impact of orthopaedic disorders, ageing, pharmacological interventions, and experimental manipulations on postural stability (4–20). Despite their broad use, considerable methodological heterogeneity exists regarding COP determination in canine research. Differences exist with respect to measurement duration, number of repetitions (4), and, importantly, measurement approach, including whether COP parameters are reported as absolute or normalised values (4–20).

While some studies assess whole-body COP using force or pressure plates positioned under all four limbs (4, 7–16, 18, 20), others derive COP parameters exclusively from the forelimbs or hindlimbs (5, 6, 8, 19). These different approaches are used interchangeably (5, 8), despite distinct biomechanical mechanisms potentially reflecting different aspects of postural stability. Whole-body COP represents the resultant ground reaction force generated by all limbs, whereas limb-specific COP measurements capture partial force distributions and may therefore be influenced by limb-pair geometry and axis-specific loading patterns. Consequently, these approaches may differentially affect conventional COP parameters such as mediolateral and craniocaudal sway.

The use of limb-specific COP measurements in canine research is largely motivated by findings from equine posturographic studies. In horses, representative stabilographic data can be obtained when animals stand with either both forelimbs or both hindlimbs on a single force plate. Furthermore, forelimb-derived COP parameters have been reported to correlate more strongly with whole-body COP measures than hindlimb-derived parameters (21). These findings have substantially informed the adoption of limb-specific approaches in canine posturography.

However, whether these methodological assumptions are valid and transferable to dogs remains unknown. It is well established that biomechanical characteristics differ between horses and dogs, as their musculoskeletal systems are adapted to different functional demands and have evolved along distinct evolutionary trajectories (22). Moreover, comparative posturographic studies have already demonstrated species-specific COP behaviour under identical experimental conditions. For example, visual deprivation increased several COP parameters, including craniocaudal and mediolateral displacement velocity, in horses (23), whereas the loss of visual input resulted in significantly decreased craniocaudal displacement and average speed in healthy adult dogs (14). However, these findings are not directly comparable, as displacement velocity and average speed represent different measures of COP dynamics.

These findings suggest that species-specific biomechanical and neuromuscular control strategies influence COP behaviour and therefore question the direct transferability of methodological concepts between species. To date, the relationship between COP data derived from whole-body and limb-specific posturography in dogs has not been investigated. A critical assessment of COP determination methods in canine static posturography is therefore warranted to improve the interpretability and comparability of postural stability outcomes.

As COP metrics reflect postural performance within a spatially defined area, their interpretation is inherently linked to the base of support (BOS), defined as the area enclosed by the limb contact points with the supporting surface (1). During quiet standing, COP adjustments act to control displacement of the center of mass within the BOS (24). Previous studies have shown that stance-related parameters contributing to the BOS, such as body width and stance width, influence COP displacement, whereas general morphometric parameters do not (25). These findings suggest that postural stability measures are influenced more strongly by BOS geometry than by overall body size.

Consequently, absolute COP parameters are expected to vary depending on individual BOS characteristics. This dependency may be particularly relevant for limb-specific measurements, which quantify postural stability within a reduced support area. Normalising COP parameters to the BOS therefore appears to be a meaningful methodological approach that may facilitate comparability between whole-body, forelimb-, and hindlimb-based assessments. Despite its conceptual relevance, BOS normalisation has rarely been applied in canine posturographic research (9–12, 14, 15).

The relevance of BOS normalisation is further illustrated by comparative posturographic studies between species with different locomotor strategies. In a recent investigation comparing postural stability in healthy humans and dogs, COP parameters were normalised to the BOS to enable meaningful comparison between bipedal and quadrupedal stance configurations. Despite the fundamental biomechanical differences between the two species, the authors reported comparable responses in mediolateral displacement and support surface parameters, suggesting that BOS normalisation can facilitate cross-species interpretation of stabilographic data (11).

Given that COP behaviour is constrained by the BOS (24), the location within the BOS at which COP is calculated may influence the resulting stabilographic parameters. Whole-body COP integrates signals from all four limbs into a single trajectory, whereas limb-specific measurements are based on only the forelimbs or hindlimbs and therefore reflect postural stability within a reduced support configuration. Consequently, measurement configuration itself may influence stabilographic outcomes.

To date, no study has systematically evaluated whether limb-specific COP measurements in dogs are associated with whole-body COP behaviour or whether BOS normalisation improves agreement between different COP measurement approaches. Therefore, the aims of the present study were (1) to examine the axis-dependent associations between whole-body, forelimb, and hindlimb COP measurements in standing dogs and (2) to evaluate the effect of BOS normalisation on agreement between COP parameters obtained using these different measurement approaches.

A two-fold hypothesis was formulated, proposing that (1) the associations between whole-body, forelimb, and hindlimb COP measurements are axis-dependent, with stronger associations in the mediolateral than in the craniocaudal direction, and (2) normalisation of COP data to the BOS improves agreement between the three measurement approaches.

## 2 Materials and methods

### 2.1 Approval and consent

This study was approved by the Ethics and Animal Welfare Committee of the University of Veterinary Medicine, Vienna in accordance with the University’s guidelines for Good Scientific Practice guidelines and national legislation (ETK-148/10/2021).

### 2.2 Animals and inclusion criteria

Initially, 41 adult pet dogs [<50% of fractional lifespan (26)] were assessed. Of these, 10 dogs were excluded because they did not meet the criteria for valid measurements, and 1 dog did not return after the initial examination, resulting in a final study population of 30 dogs. The inclusion criteria consisted of the absence of any clinical orthopaedic, neurological, or visual diseases, and body mass of ≥10 kg. All dogs underwent a general clinical examination including visual gait assessment, orthopaedic and neurologic examination. To exclude dogs with unrecognized lameness or gait abnormalities not apparent during clinical examination, objective gait analysis was performed using a pressure measurement plate (FDM Type 2, Zebris Medical GmbH, Allgäu, Germany). Only dogs exhibiting symmetrical gait patterns, defined by a symmetry index of peak vertical force and vertical impulse <3% (27), were included in the study. Gait analysis was conducted as described previously (4, 14).

The study population consisted of dogs of various breeds. The most frequently represented breeds were Labrador Retrievers (*n* = 6), Border Collies (*n* = 5) and mixed-breed dogs (*n* = 3). All other breeds were represented by one or two individuals, including Belgian Malinois, Large Poodle, Flat-Coated Retriever, Beagle, Tamaskan, Irish Setter, Golden Retriever, Husky, Magyar Vizsla, Greyhound, Australian Shepherd, English Springer Spaniel, Pointer, Pinscher, and Coonhound. The mean age, body mass and withers height (measured from the dorsal border of the scapula) was 3.37 ± 1.53 years, 22.87 ± 6.57 kg and 55.28 ± 7.30 cm, respectively.

### 2.3 Posturography

Static posturography was performed using a pressure measurement plate (FDM Type 2, Zebris Medical GmbH, Allgäu, Germany) equipped with 15,360 sensors covering an area of 203 × 54.2 cm and a sampling frequency of 100 Hz. The sensor size of the platforms was 0.72 × 0.72 cm. The pressure plate was covered with a black 1-mm-thick non-slip rubber mat. All measurement procedures were filmed using a Panasonic NV-MX500 camera (Panasonic, Kadoma, Osaka, Japan).

The pet owner led the dog onto the pressure measurement plate and halted it in a straight and square position. During data collection, the pet owner stood in front of the dog to discourage further movement and maintain the animal’s attention. Each trial lasted for a minimum of 30 s and was repeated as necessary until at least two valid data segments of 5 s duration were obtained for each dog (4). A valid measurement was defined as a quiet stance without any movement of the head and paws.

### 2.4 Data analysis

For each dog, the first 2 valid 5 s data segments of quiet standing were selected for analysis, in accordance with previously established methodology (4). Segment validity was determined by visual assessment of the measurement, with valid segments showing no observable movements or postural adjustments and minimal COP fluctuations. Each segment was subsequently analysed using three different approaches to COP determination: (1) whole-body COP, based on the combined contribution of all 4 paw contact points; (2) forelimb COP, based exclusively on the forelimb contact points; and (3) hindlimb COP, based exclusively on the hindlimb contact points. For each dog and COP measurement approach, the values derived from the 2 selected 5 s segments were averaged before statistical analysis (4).

To reduce high-frequency noise while preserving physiologically relevant components of postural sway, COP data were low-pass filtered using a cut-off frequency of 6 Hz (15).

### 2.5 Parameters under investigation

The following parameters were used for objective gait analysis during walk:

- Peak vertical force (PFz in Newton (N)).
- Vertical impulse (IFz in N/s), describing the impulse in the Z-direction.
- Additionally, a symmetry index (SI%) was calculated as a percentage for both parameters using the following modified equation (28, 29):

$$\text{SIXFz}\;(\%)=\mathrm{abs}\;(\frac{({\mathrm{XFz}}_{\mathrm{LLx}}-{\mathrm{XFz}}_{\mathrm{RLx}}\;}{({\mathrm{XFz}}_{\mathrm{LLx}}+{\mathrm{XFz}}_{\mathrm{RLx})}})x\;100$$

Where XFz represents the mean PFz or IFz value of the valid steps. LLx and RLx denote the left and right limb, respectively, with Lx referring to either the forelimbs or the hindlimbs.

**Figure 1:**
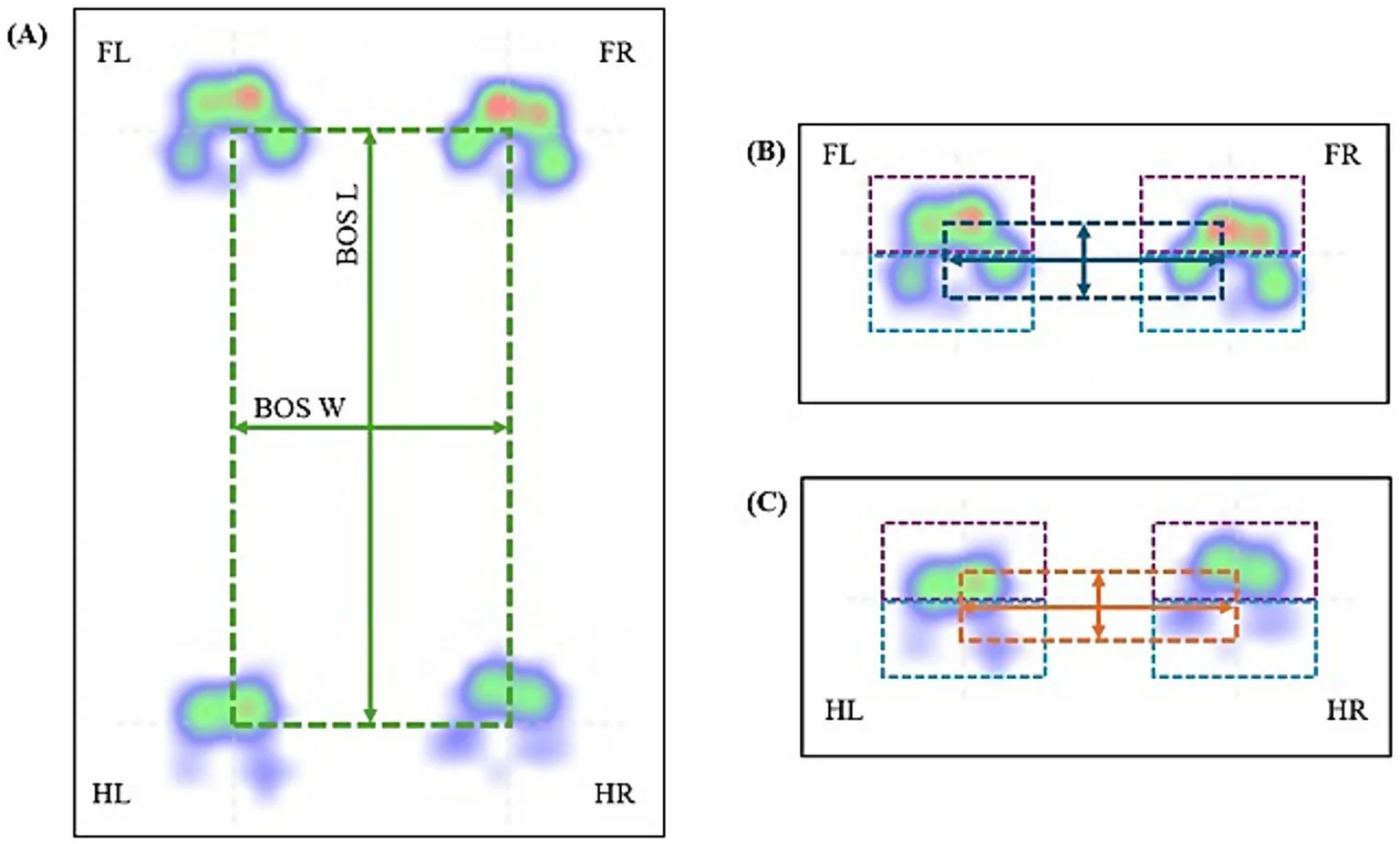
Schematic demonstration of the calculation of the base of support (BOS) of **(A)** the whole body (green dotted rectangle), **(B)** the forelimbs (dark blue dotted rectangle), and **(C)** the hindlimbs (orange dotted rectangle). For limb-specific measurements, the paw contact areas were subdivided into a cranial (purple dotted rectangle) and a caudal (light blue dotted rectangle) section. BOS L, BOS length; BOS W, BOS width; FL, Forelimb left; FR, Forelimb right; HL, Hindlimb left; HR, Hindlimb right; graphic adapted from previous research (11).

Base of support (Figure 1):

- Base of support (BOS): Area enclosed by the coordinates of the centres of the paw contact points, expressed in cm^2^. For limb-specific measurements, the BOS was calculated based on the corresponding forelimb or hindlimb paw contact points.
- Base of support length (BOS L): Craniocaudal distance between the midpoint of the centres of the left and right forelimbs and the midpoint of the centres of the left and right hindlimbs, expressed in cm. For limb-specific measurements, paw contact areas were subdivided into cranial and caudal sections. BOS L was defined as the craniocaudal distance between the midpoint of the cranial and caudal paw sections of the respective limb pair.
- Base of support width (BOS W): Mediolateral distance between the centres of the left and right limbs, expressed in cm. For limb-specific measurements, paw contact areas were subdivided into cranial and caudal sections. BOS W was calculated as the distance between the centres of the left and right paw sections within the same cranial or caudal region.

Center of pressure:

- Craniocaudal displacement (CCD): Mean deviation on the craniocaudal axis in mm was normalised to the BOS L and expressed as a percentage (CCD%).
- Mediolateral displacement (MLD): Mean deviation on the lateral axis in mm was normalised to the BOS W and expressed as a percentage (MLD%).
- Statokinesiogram length (L): the length of the line that joins the points of the COP trajectory in m was normalised to the BOS and expressed as a percentage (L%).
- Support surface or statokinesiogram (SS): The area determined by an ellipse that contains 90% of the points of the COP trajectory in mm^2^ was normalised to the BOS and expressed as a percentage (SS%).
- Average speed (AS) of COP sway in mm/s.

### 2.6 Statistical analysis

All statistical analyses were performed using IBM SPSS Statistics (version 29; IBM Corp., Armonk, NY, United States). Data distribution was assessed using the Shapiro–Wilk test for each COP parameter within each measurement condition. As normality assumptions were met for only 59% of the datasets, non-parametric statistical methods were applied to ensure robust inference.

Associations between COP parameters obtained under different measurement conditions (whole-body, forelimb, and hindlimb measurements) were evaluated using Spearman’s rank correlation coefficients. Correlation analyses were conducted pairwise between measurement conditions for each COP parameter. Correlation strength was interpreted as weak (*ρ* = 0.2–0.39), moderate (0.4–0.59), and strong (≥0.6). Corresponding two-tailed *p*-values were computed for each correlation. Given the exploratory nature of the study and the number of multiple comparisons performed, the Benjamini-Hochberg procedure was applied to control the false discovery rate (FDR). Adjusted *p*-values were used to determine statistical significance. Spearman’s rank correlation coefficients were interpreted as measures of association between measurement approaches, reflecting the preservation of relative ranking rather than agreement between numerical values.

Agreement between measurement approaches was further assessed using Bland–Altman analysis. For each pairwise comparison, the mean difference (bias) between measurement approaches was calculated together with the limits of agreement (LoA), defined as the bias ± 1.96 standard deviations (SD) of the differences. For each individual, the mean of the two measurements was plotted against the difference between the measurements. In addition, 95% confidence intervals (CI) for the bias were calculated as bias ± t × (SD/√*n*), where *n* denotes the sample size and t represents the critical value of the t-distribution. Approximate 95% CIs for the lower and upper LoA were calculated as LoA ± t × SE_LoA_, where SE_LoA_ was calculated as SD × √[1/n + 1.96^2^/(2(*n* − 1))]. Bland–Altman plots were visually inspected to evaluate the presence of systematic and proportional bias and to assess the agreement between measurement approaches across the measurement range.

All statistical tests were two-tailed, and statistical significance was set at an alpha level of *p* < 0.05.

## 3 Results

### 3.1 Non-normalised COP parameters

Descriptive statistics for non-normalised parameters and measurement conditions are presented in Table 1. The Spearman’s rank correlation analysis revealed positive correlations between the different measurement approaches for all evaluated non-normalised parameters (CCD, MLD, SS, L, and AS), with correlation strengths ranging from weak to strong (Table 2).

**Table 1:** Descriptive statistics (median (Q1–Q3)) of the different measurement conditions (C) for non-normalised COP parameters including craniocaudal displacement (CCD), mediolateral displacement (MLD), support surface (SS), statokinesiogram length (L) and average speed (AS).

C	CCD (mm)	MLD (mm)	SS (mm^2^)	L (m)	AS (mm/s)
WB	5.40 (4.79–6.36)	2.20 (1.82–2.81)	6.50 (4.81–10.77)	0.06 (0.05–0.08)	11.90 (10.22–15.76)
FL	0.72 (0.57–1.06)	2.35 (1.83–3.01)	0.76 (0.56–1.10)	0.02 (0.02–0.03)	3.79 (3.19–5.15)
HL	0.85 (0.62–1.20)	3.54 (2.65–4.60)	1.56 (0.84–2.24)	0.03 (0.02–0.04)	5.95 (4.89–8.08)

FL, forelimb; HL, hindlimb; WB, whole body.

**Table 2:** Spearman’s rho correlation and Benjamini-Hochberg adjusted *p*-values (p_adj) between measurement approaches CI and CII (condition I and condition II) for non-normalised COP parameters for craniocaudal displacement (CCD), mediolateral displacement (MLD), support surface (SS), statokinesiogram length (L) and average speed (AS).

C I	C II	CCD	p_adj	MLD	p_adj	SS	p_adj	L	p_adj	AS	p_adj
WB	FL	0.30	0.098	**0.75**	<0.0001*	**0.69**	<0.0001*	**0.77**	<0.0001*	**0.77**	<0.0001*
WB	HL	*0.44*	0.020*	**0.65**	<0.0001*	**0.73**	<0.0001*	**0.69**	<0.0001*	**0.69**	<0.0001*
FL	HL	*0.42*	0.023*	*0.40*	0.033*	*0.44*	0.020*	**0.69**	<0.0001*	**0.69**	<0.0001*

FL, forelimb; HL, hindlimb; WB, whole body; bold font indicates a strong correlation; italic font indicates a moderate correlation; *Statistically significant.

Between whole-body and forelimb measurements, CCD showed a weak positive correlation that was not statistically significant. In contrast, MLD, L, and AS demonstrated strong positive correlations, while SS showed a moderate positive correlation; all these correlations were statistically significant.

For whole-body and hindlimb measurements, CCD, MLD, L, and AS showed moderate positive correlations, while SS demonstrated a strong positive correlation. All correlations in this comparison were statistically significant.

Comparisons between forelimb and hindlimb measurements revealed significant moderate positive correlations across all parameters.

**Figure 2:**
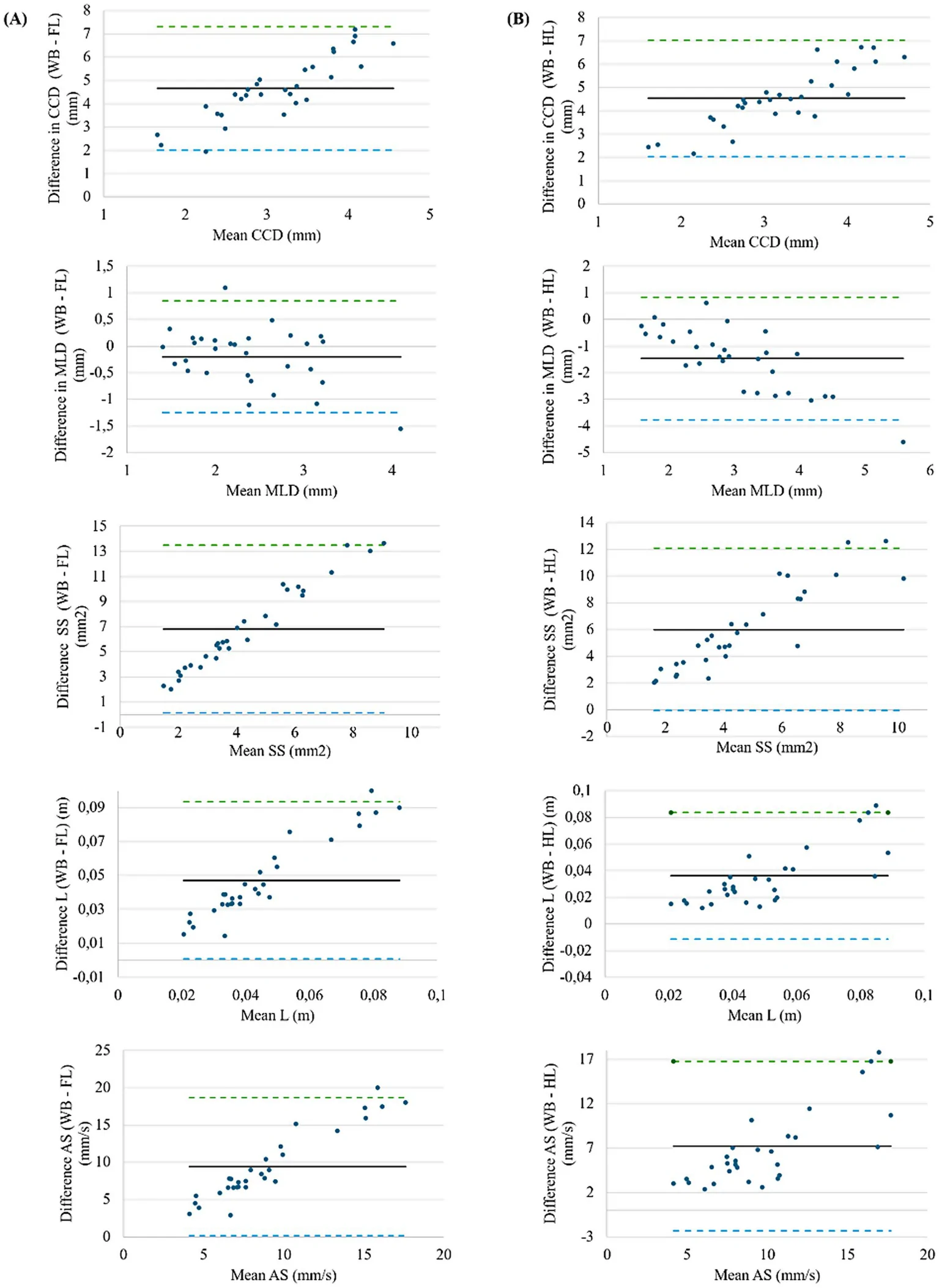
Bland–Altman plots assessing agreement between posturographic measurement approaches for non-normalised center of pressure (COP) parameters. Panel **(A)** shows comparisons between whole-body (WB) and forelimb (FL) measurements, whereas Panel **(B)** shows comparisons between whole-body (WB) and hindlimb (HL) measurements. Craniocaudal displacement (CCD), mediolateral displacement (MLD), support surface (SS), statokinesiogram length (L), and average speed (AS) are presented. The solid black line represents the mean bias, while the upper and lower dashed lines indicate the limits of agreement (LoA; bias ± 1.96 standard deviations of the differences). Positive values indicate larger parameter values for the WB measurements compared to **(A)** FL or **(B)** HL.

Bland–Altman analysis results for the non-normalised COP parameters, including mean bias and the lower and upper LoA with their respective 95% confidence intervals, are presented in Table 3 and Figure 2. Agreement between measurement approaches varied across parameters, with both systematic bias and the width of the LoA differing between comparisons.

**Table 3:** Bland–Altman analysis of agreement between measurement approaches for non-normalised center of pressure (COP) parameters.

Comparison	Parameter	Bias (95% CI)	Lower LoA (95% CI)	Upper LoA (95% CI)
WB vs. FL	CCD (mm)	4.66 (4.15 to 5.16)	2.01 (1.14 to 2.88)	7.31 (6.44 to 8.18)
	MLD (mm)	−0.20 (−0.40 to 0.00)	−1.25 (−1.60 to −0.91)	0.85 (0.50 to 1.20)
	SS (mm^2^)	6.80 (5.53 to 8.07)	0.13 (−2.06 to 2.33)	13.46 (11.27 to 15.65)
	L (m)	0.05 (0.04 to 0.06)	0.00 (−0.01 to 0.02)	0.09 (0.08 to 0.11)
	AS (mm/s)	9.41 (7.65 to 11.18)	0.15 (−2.90 to 3.20)	18.67 (15.62 to 21.72)
WB vs. HL	CCD (mm)	4.53 (4.06 to 5.01)	2.04 (1.22 to 2.86)	7.03 (6.21 to 7.85)
	MLD (mm)	−1.47 (−1.91 to −1.03)	−3.78 (−4.54 to −3.02)	0.83 (0.07 to 1.59)
	SS (mm^2^)	6.01 (4.85 to 7.17)	−0.06 (−2.06 to 1.94)	12.08 (10.08 to 14.08)
	L (m)	0.04 (0.03 to 0.05)	−0.01 (−0.03 to 0.00)	0.08 (0.07 to 0.10)
	AS (mm/s)	7.22 (5.40 to 9.03)	−2.31 (−5.45 to 0.83)	16.74 (13.61 to 19.88)

Mean bias, 95% confidence intervals (95% CI) of the bias, and lower and upper limits of agreement (LoA) with their respective 95% CIs are presented for comparisons between whole-body (WB), forelimb (FL), and hindlimb (HL) measurement approaches. Non-normalised COP parameters include craniocaudal displacement (CCD), mediolateral displacement (MLD), statokinesiogram length (L), average speed (AS), and support surface (SS). Bias was calculated as the mean difference between measurement approaches, and LoA were calculated as bias ± 1.96 standard deviations of the differences.

For comparisons between whole-body and forelimb measurements, CCD, SS, L, and AS showed positive systematic biases, whereas MLD exhibited a small negative bias (Table 3). Visual inspection of the Bland–Altman plots suggested increasing differences with increasing parameter magnitude for CCD, SS, L, and AS, indicating the presence of proportional bias. In contrast, MLD showed no obvious proportional trend (Figure 2A).

For comparisons between whole-body and hindlimb measurements, positive systematic biases were observed for CCD, SS, L, and AS, whereas MLD demonstrated a larger negative bias than observed for the whole-body versus forelimb comparison (Table 3). Like the whole-body versus forelimb comparison, proportional bias was evident for CCD, SS, L, and AS, while MLD displayed comparatively homogeneous differences across the measurement range (Figure 2B).

### 3.2 Normalised COP parameters

Descriptive statistics for the normalised parameters across measurement conditions are presented in Table 4. The Spearman’s rank correlation coefficient analysis of the normalised COP parameters (CCD%, MLD%, SS%, and L%) revealed statistically significant positive correlations between the different measurement approaches, ranging from weak to strong (Table 5).

**Table 4:** Descriptive statistics (median (Q1–Q3)) of the different measurement conditions (C) for normalised COP parameters including craniocaudal displacement (CCD%), mediolateral displacement (MLD%), support surface (SS%) and statokinesiogram length (L%).

C	CCD%	MLD%	SS%	L%
WB	1.01 (0.82–1.21)	1.19 (0.92–1.37)	0.08 (0.05–0.09)	0.06 (0.04–0.08)
FL	1.70 (1.24–2.73)	1.45 (1.05–1.72)	0.12 (0.09–0.19)	0.28 (0.20–0.38)
HL	2.54 (1.87–3.54)	1.70 (1.21–1.98)	0.20 (0.13–0.27)	0.42 (0.35–0.46)

FL, forelimb; HL, hindlimb; WB, whole body.

**Table 5:** Spearman’s rho correlation and Benjamini-Hochberg adjusted *p*-values between measurement approaches CI and CII (condition I and condition II) for normalised COP parameters for craniocaudal displacement (CCD%), mediolateral displacement (MLD%), support surface (SS%) and statokinesiogram length (L%).

C I	C II	CCD%	p_adj	MLD%	p_adj	SS%	p_adj	L%	p_adj
WB	FL	*0.40*	0.032*	**0.66**	<0.0001*	**0.68**	<0.0001*	**0.79**	<0.0001*
WB	HL	*0.48*	0.012*	*0.59*	0.001*	**0.66**	<0.0001*	**0.63**	<0.0001*
FL	HL	*0.45*	0.017*	0.31	0.098	*0.45*	0.017*	**0.68**	<0.0001*

FL, forelimb; HL, hindlimb; WB, whole body; bold font indicates a strong correlation; italic font indicates a moderate correlation; *Statistically significant.

Between whole-body and forelimb measurements, CCD%, MLD% and SS% showed moderate positive correlations that were statistically significant. L% showed a statistically significant strong positive correlation. For whole-body and hindlimb measurements, all normalised COP parameters revealed a significant moderate correlation.

Comparisons between forelimb and hindlimb measurements revealed significant moderate positive correlations across all parameters, except MLD%.

**Figure 3:**
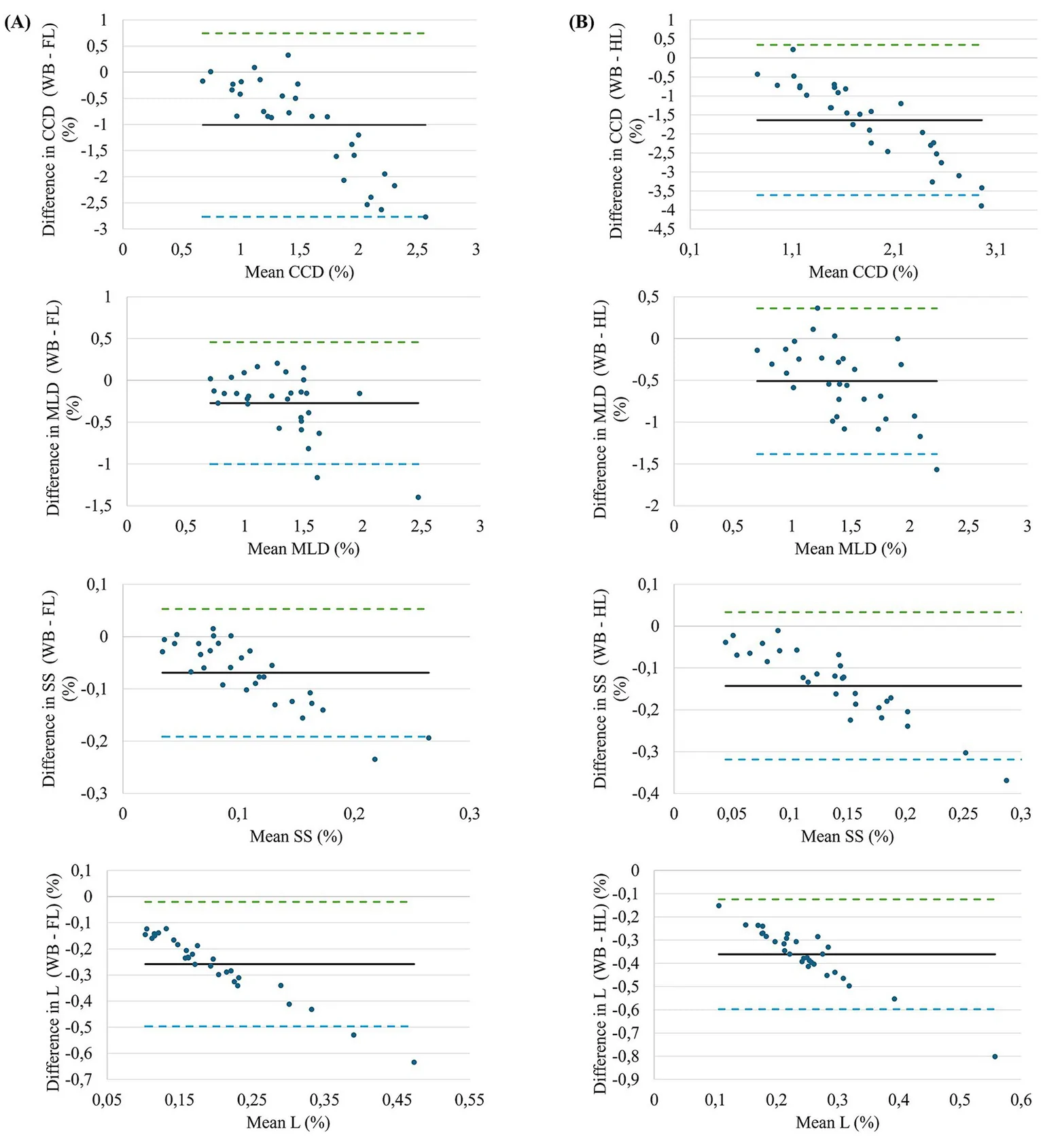
Bland–Altman plots assessing agreement between posturographic measurement approaches for normalised center of pressure (COP) parameters. Panel **(A)** shows comparisons between whole-body (WB) and forelimb (FL) measurements, whereas Panel **(B)** shows comparisons between whole-body (WB) and hindlimb (HL) measurements. Craniocaudal displacement (CCD%), mediolateral displacement (MLD%), support surface (SS%), and statokinesiogram length (L%) are presented. The solid black line represents the mean bias, while the upper and lower dashed lines indicate the limits of agreement (LoA; bias ± 1.96 standard deviations of the differences). Positive values indicate greater parameter values obtained from the whole-body measurement approach relative to the respective limb-specific measurement approach.

Bland–Altman analysis results for the normalised COP parameters, including mean bias and the lower and upper LoA with their respective 95% confidence intervals, are presented in Table 6 and Figure 3. Agreement between measurement approaches varied across parameters, with systematic bias and the width of the LoA differing between comparisons.

**Table 6:** Bland–Altman analysis of agreement between measurement approaches for normalised center of pressure (COP) parameters.

Comparison	Parameter	Bias (95% CI)	Lower LoA (95% CI)	Upper LoA (95% CI)
WB vs. FL	CCD (%)	−1.01 (−1.35 to −0.68)	−2.76 (−3.34 to −2.19)	0.74 (0.17 to 1.32)
	MLD (%)	−0.27 (−0.41 to −0.13)	−1.00 (−1.24 to −0.76)	0.46 (0.22 to 0.70)
	SS (%)	−0.07 (−0.09 to −0.05)	−0.19 (−0.23 to −0.15)	0.05 (0.01 to 0.09)
	L (%)	−0.26 (−0.30 to −0.21)	−0.50 (−0.58 to −0.42)	−0.02 (−0.10 to 0.06)
WB vs. HL	CCD (%)	−1.64 (−2.01 to −1.26)	−3.61 (−4.26 to −2.96)	0.34 (−0.31 to 0.99)
	MLD (%)	−0.51 (−0.68 to −0.34)	−1.38 (−1.67 to −1.09)	0.36 (0.07 to 0.65)
	SS (%)	−0.14 (−0.18 to −0.11)	−0.32 (−0.38 to −0.26)	0.03 (−0.02 to 0.09)
	L (%)	−0.36 (−0.41 to −0.32)	−0.60 (−0.68 to −0.52)	−0.12 (−0.20 to −0.05)

Mean bias, 95% confidence intervals (95% CI) of the bias, and limits of agreement (LoA) with their respective 95% CIs are presented for comparisons between whole-body (WB), forelimb (FL), and hindlimb (HL) measurement approaches. Normalised COP parameters include craniocaudal displacement (CCD%), mediolateral displacement (MLD%), support surface (SS%), and statokinesiogram length (L%). Bias was calculated as the mean difference between measurement approaches, and LoA were calculated as bias ± 1.96 standard deviations of the differences.

For comparisons between whole-body and forelimb measurements, all normalised COP parameters exhibited negative systematic biases, indicating lower parameter values obtained from the whole-body measurement approach than from the forelimb approach (Table 6). The smallest bias was observed for SS%, whereas CCD% and L% showed the largest systematic differences. Visual inspection of the Bland–Altman plots suggested proportional bias for CCD%, SS%, and particularly L%, with differences increasing as the mean parameter values increased. In contrast, MLD% demonstrated comparatively homogeneous differences across the measurement range (Figure 3A).

For comparisons between whole-body and hindlimb measurements, all parameters likewise exhibited negative systematic biases (Table 6). The largest bias was observed for CCD%, whereas SS% showed the smallest systematic difference. Similar to the whole-body versus forelimb comparison, proportional bias was evident for CCD%, SS%, and L%, while MLD% displayed less pronounced trends across the measurement range (Figure 3B).

## 4 Discussion

The aims of this study were to examine the associations between whole-body, forelimb-, and hindlimb-based COP measurements in standing dogs and to evaluate the effect of BOS normalisation on the agreement of COP parameters obtained using these different measurement approaches. It was hypothesised that (1) the associations between whole-body, forelimb, and hindlimb COP measurements would be axis-dependent, with stronger associations for mediolateral than craniocaudal displacement, and (2) normalisation of COP parameters to the BOS would improve agreement between measurement approaches. Overall, the findings partially supported these hypotheses.

Regarding the first hypothesis, the results demonstrate that the associations between whole-body, forelimb, and hindlimb COP measurements are parameter-dependent and differ between the craniocaudal and mediolateral axes. MLD showed stronger correlations between whole-body and both limb-specific measurement approaches than CCD. Comparisons between forelimb and hindlimb measurements for MLD revealed only moderate correlations, suggesting that mediolateral COP measures may allow only cautious extrapolation between measurement approaches. In contrast, CCD generally demonstrated weaker associations, with only moderate correlations for the non-normalised data and no significant correlation between whole-body and forelimb measurements. Hindlimb-derived CCD measurements consistently showed stronger associations with whole-body measurements than forelimb-derived values, suggesting that hindlimb-derived measurements may more closely reflect patterns of whole-body COP behaviour in the craniocaudal direction.

For L and AS, strong correlations were observed across all measurement approaches. Similarly, SS demonstrated strong correlations between whole-body and limb-specific measurements and moderate correlations between forelimb and hindlimb measurements. While these findings indicate strong associations between measurement approaches, the Bland–Altman analyses revealed systematic differences between approaches. Visual inspection of the Bland–Altman plots further indicated magnitude-dependent differences for several parameters. In particular, the differences in CCD, SS, L, and AS tended to increase with increasing parameter magnitude. MLD showed comparatively small systematic differences for the whole-body versus forelimb comparison, whereas a larger negative bias was observed for the whole-body versus hindlimb comparison. Thus, despite the strong associations observed for several COP parameters, the systematic and magnitude-dependent differences between measurement approaches indicate that whole-body, forelimb, and hindlimb posturographic measurements cannot be considered directly interchangeable.

These findings differ from previous posturographic studies in horses, in which stabilographic parameters obtained from either forelimb or hindlimb measurements closely approximated whole-body COP measures, with forelimb-derived parameters showing particularly strong association (21). Based on these observations, limb-specific COP measurements, especially forelimb-based approaches, have been adopted in canine posturographic studies as proxies for whole-body measurements (5, 6, 8, 19). However, the equine study evaluated associations between measurement approaches using correlation and regression analyses only, without formally assessing agreement between methods (21). In the present study, the combination of correlation and Bland–Altman analyses demonstrated that moderate to strong correlations do not necessarily imply agreement or interchangeability, as several COP parameters exhibited systematic and proportional bias despite significant associations. Consequently, the present results indicate that associations between whole-body and limb-specific COP measurements in dogs are more variable and parameter-dependent than previously assumed. Methodological assumptions derived from equine posturography should therefore not be transferred directly to canine studies without species-specific validation.

These interspecies differences are likely explained by anatomical and biomechanical characteristics. Horses possess a relatively rigid limb configuration and a more constrained postural strategy during quiet standing, whereas dogs exhibit greater joint mobility and more flexible postural adjustments. The greater range of motion of the canine shoulder and hip joints may facilitate compensatory movements that redistribute body sway between limb pairs, thereby reducing the extent to which forelimb- or hindlimb-derived COP measurements represent whole-body COP behaviour (13, 22). Furthermore, the digitigrade posture and breed-related variation in paw morphology and pressure distribution may additionally influence how postural adjustments are distributed across the limbs (30–32). Collectively, these biomechanical differences may explain why associations between limb-specific and whole-body COP measurements observed in horses cannot be directly extrapolated to dogs.

The second hypothesis proposed that BOS normalisation would improve agreement between measurement approaches. This hypothesis was only partially supported. Normalisation strengthened the association between whole-body and forelimb CCD measurements, converting a weak, non-significant association into a significant moderate correlation. However, for most parameters, correlation strength changed only marginally following normalisation. More importantly, Bland–Altman analyses demonstrated that systematic bias persisted after normalisation, and proportional bias remained evident for several parameters. Thus, although BOS normalisation standardises COP excursions relative to the available support area (11), it does not eliminate methodological differences between whole-body and limb-specific measurements.

This finding is biomechanically plausible because normalisation modifies only the spatial scale of COP excursions, whereas the underlying force distribution remains fundamentally different between measurement approaches. Whole-body COP represents the resultant ground reaction force generated by all four limbs, whereas forelimb- and hindlimb-derived COP trajectories represent only partial force distributions within reduced support configurations. Consequently, even after BOS normalisation, these measurement approaches capture different components of postural control and therefore cannot be expected to yield equivalent measures of postural stability.

An additional finding was that the magnitude of postural sway depended on the selected measurement approach. For several non-normalised parameters, forelimb-derived measurements yielded smaller COP excursions than whole-body or hindlimb measurements, suggesting greater forelimb stability during quiet standing. This observation is consistent with the approximately 60% weight distribution borne by the thoracic limbs in dogs (33). Following BOS normalisation, however, whole-body measurements frequently exhibited the smallest relative sway values. This likely reflects the different reference areas used for normalisation, as whole-body COP was expressed relative to the BOS defined by all four limbs, whereas limb-specific COP parameters were normalised to the smaller BOS of either the forelimbs or hindlimbs. Consequently, identical absolute COP excursions represent smaller relative values for whole-body measurements.

These findings have important implications for the interpretation of stabilographic data across studies using different posturographic methodologies. Because whole-body and limb-specific measurements quantify COP behaviour within different biomechanical contexts, results obtained using different measurement approaches should not be directly compared without accounting for these methodological differences. This issue is particularly relevant for studies investigating orthopaedic disorders. Previous investigations comparing dogs with elbow dysplasia and cranial cruciate ligament rupture reported larger SS in dogs with forelimb disease than in dogs with hindlimb disease, although forelimb pathology was evaluated using forelimb measurements and hindlimb pathology using hindlimb measurements (5). In the present study, hindlimb-derived SS values were systematically larger than forelimb-derived values in healthy dogs, while the association between the 2 limb-specific approaches was only moderate. Together with the systematic differences identified by Bland–Altman analysis, these findings raise the possibility that some previously reported differences in SS between forelimb and hindlimb disease groups may, at least in part, reflect differences in measurement approach rather than disease-related effects alone. This interpretation remains speculative, as the present study included only healthy dogs, and should therefore be investigated in clinical populations.

A similar methodological influence may also contribute to findings from experimental balance assessments using dynamic platforms. Significantly larger SS, L, and average velocity were reported when dogs stood with only the forelimbs on a perturbation platform than during static standing, whereas perturbations involving all four limbs resulted in the greatest values (8). It should be noted that average velocity, as reported in that study, differs from AS assessed in the present study; COP velocity was not evaluated in the present analysis. Although these findings were attributed primarily to the increasing balance challenge, the present results raise the possibility that differences in measurement approach may also have contributed to the observed differences between the static and forelimb-based conditions. Consequently, methodological effects should be considered when interpreting stabilographic outcomes obtained under different support configurations.

Several limitations should be considered. The relatively small and young study population, exclusion of dogs weighing <10 kg, and heterogeneity in breeds and body conformations may limit the generalisability of the findings. Orthopaedic health was assessed by clinical examination and objective gait analysis using a pressure plate, but without diagnostic imaging; thus, subclinical structural abnormalities cannot be excluded (34). The analysis was based on the mean of the first 2 valid 5 s segments per dog, in accordance with previously established methodology (4), with identical segments used for all COP approaches. Nevertheless, the short observation periods and potential effects of subtle head and neck position changes or attention towards the owner should be considered. Furthermore, the limb-specific BOS requires further validation, and the use of a single measurement system and laboratory may limit generalisability. The relatively small sample size may also have limited the precision of the Bland–Altman estimates. As no predefined thresholds for acceptable agreement were established, the LoA should be interpreted descriptively. Future studies should include larger and more diverse populations and evaluate COP measures under more challenging posturographic conditions.

## 5 Conclusion

This study demonstrates that whole-body, forelimb-, and hindlimb-based COP measurements in standing dogs are correlated but not directly interchangeable. While mediolateral COP parameters generally showed stronger associations between measurement approaches than craniocaudal parameters, Bland–Altman analyses revealed systematic differences and, for several variables, magnitude-dependent patterns, indicating limited agreement despite significant correlations. BOS normalisation altered the magnitude of several COP parameters but did not consistently improve agreement or eliminate methodological differences between measurement approaches. These findings highlight that measurement configuration substantially influences stabilographic outcomes and should therefore be carefully considered when interpreting posturographic data and comparing results across studies.

## Data Availability

The original contributions presented in the study are included in the article/supplementary material, further inquiries can be directed to the corresponding author.
